# REIMAGINING END OF LIFE: ALTERNATIVE APPROACHES TO SERIOUS ILLNESS CARE CONVERSATIONS

**DOI:** 10.1093/geroni/igac059.1809

**Published:** 2022-12-20

**Authors:** Maresi Berry-Stoelzle, Cristina Ortiz, Jessica Trepanier, Sarah Murray, Melissa Steffen, Bruce Alexander, Kenda Stewart Steffensmeier

**Affiliations:** Iowa City VA HCS, Iowa City, Iowa, United States; University of Iowa VA, Iowa City, Iowa, United States; Iowa City VA HCS, Iowa City, Iowa, United States; Iowa City VA HCS, Iowa City, Iowa, United States; Iowa City VA HCS, Iowa City, Iowa, United States; Iowa City VA Health Care System, Iowa City, Iowa, United States; Iowa City VA HCS, Iowa City, Iowa, United States

## Abstract

Serious illness care conversations are intended to document and protect patients’ medical treatment wishes. In the outpatient management of chronic diseases, it has been difficult to pinpoint ideal timing for these conversations. The goal of this project is to analyze current practice within a VA health care system for identifying and referring veterans for palliative care services in the last years of life. To develop a targeted intervention, we relied on different forms of information to identify life events which should trigger a goals of care conversation, possibly even a formal palliative care referral. This study describes how we operationalized knowledge gained from: 1) Veteran community members via a project-dedicated engagement panel, 2) semi-structured interviews with providers, and 3) review of patients’ clinic notes written by clinicians during the last 5 years of life. Tapping into the knowledge and experience of multiple parties provided nuance and complexity to our approach to intervention development. From the Veteran community member engagement panel, we learned that trusting relationships were essential. Providers emphasized that having enough time during appointments and timing of the conversation during a patient’s disease trajectory were important. Finally, chart review indicated a need for broaching serious illness conversations early and throughout the duration of their care. The goal of an earlier intervention is to engage veterans when they are medically stable and prior to prolonged hospitalization. These findings structured our ongoing intervention that focuses on identifying and reaching out to Veterans with serious illnesses.

